# Sub-lethal concentration of a colloidal nanosilver formulation (Silversol®) triggers dysregulation of iron homeostasis and nitrogen metabolism in multidrug resistant *Pseudomonas aeruginosa*

**DOI:** 10.1186/s12866-023-03062-x

**Published:** 2023-10-23

**Authors:** Gemini Gajera, Nidhi Thakkar, Chhaya Godse, Anselm DeSouza, Dilip Mehta, Vijay Kothari

**Affiliations:** 1 Institute of Science, Nirma University, Ahmedabad, 382481 India; 2 Viridis BioPharma Pvt Ltd, Mumbai, India

**Keywords:** AMR (antimicrobial resistance); *Pseudomonas aeruginosa*, Silver, Transcriptome, Nitrosative stress, Iron homeostasis

## Abstract

**Background:**

*Pseudomonas aeruginosa* is a notorious pathogen. Its multidrug resistant strains are listed among priority pathogens against whom discovery of novel antibacterial agents and, elucidation of new anti-pathogenicity mechanisms are urgently warranted. This study describes multiple antibacterial effects of a colloidal nano-silver formulation- Silversol® against a multi-drug resistant strain of *P. aeruginosa*.

**Results:**

Minimum inhibitory concentration (MIC) of Silversol® against *P. aeruginosa* was found to be 1.5 ppm; and at sub-MIC of 1 ppm, it was able to alter quorum-sensing regulated pigmentation (pyocanin 82%↓; pyoverdine 48%↑), exopolysaccharide synthesis (76%↑) and biofilm formation, susceptibility to antibiotics (streptomycin and augmentin), protein synthesis and export (65%↑), nitrogen metabolism (37%↑ nitrite accumulation), and siderophore production in this pathogen. Network analysis of the differentially expressed genes in the transcriptome of the silversol-treated bacterium identified ten genes as the potential molecular targets: norB, norD, nirS, nirF, nirM, nirQ, nosZ, nosY, narK1, and norE (all associated with nitrogen metabolism or denitrification). Three of them (norB, narK1, and norE) were also validated through RT-PCR.

**Conclusions:**

Generation of nitrosative stress and disturbance of iron homeostasis were found to be the major mechanisms associated with anti-*Pseudomonas* activity of Silversol®.

**Supplementary Information:**

The online version contains supplementary material available at 10.1186/s12866-023-03062-x.

## Introduction

Silver has a long history of therapeutic use in traditional medicine. In the modern era too, many reports [1–3] have been published describing various biological activities of silver. Antimicrobial activity of different forms of silver are also well known e.g., antibacterial [4, 5], antifungal [6], antiviral [7], antiprotozoal [8], and anthelmintic [9]. Different forms of silver (e.g., metallic, colloidal, silver salts) may have different modes of action, and varying degree of biological activity. Antibacterial activity of silver becomes even more important in face of the resistance displayed by bacterial pathogens against conventional antibiotics. Till now, there is not much indication from literature about bacteria developing resistance against silver. In general, silver is believed to be killing bacteria by altering cell membrane permeability, generating reactive oxygen species (ROS), and interrupting DNA replication [10–14]. However, much investigation still is required to elucidate precise molecular mechanisms associated with antibacterial activity of silver.

Since metallic form of silver is known to be more potent than its ionic form [15], we chose a solution of colloidal silver for the purpose of this study. Target pathogen for this study was *Pseudomonas aeruginosa*, one of the most notorious and versatile bacterial pathogens. Its antibiotic-resistant strains have been listed by Centers for Disease Control and Prevention (CDC; https://www.cdc.gov/drugresistance/biggest-threats.html), World Health Organization (WHO; https://www.who.int/publications/i/item/WHO-EMP-IAU-2017.12), and Department of Biotechnology of the Indian government (DBT; https://dbtindia.gov.in/sites/default/files/IPPL_final.pdf) among priority pathogens, against whom there is an urgent need to discover new antibiotics [16]. This study investigated effect of Silversol® (a colloidal nano-silver formulation) on *P. aeruginosa*’s growth, various virulence traits, and gene expression profile at the whole transcriptome level. Silversol is being used primarily for multiple therapeutic applications e.g. management of oral health [17], skin diseases, burns, diabetic ulcer, and wound-care/ wound-disinfection (https://www.rxsilver.com/index_htm_files/ABLSilversafety.pdf). *P. aeruginosa* being one of the common wound-isolates [18, 19], we attempted to gain insight into Silversol’s antibacterial activity against this pathogen.

## Methods

### Colloidal silver formulation

The test formulation Silversol® (32 ppm; Batch number: Sil Water/32P/19–61) originally developed by American Biotech Labs (USA) was procured from Viridis BioPharma Pvt Ltd, Mumbai, India. It is a colloidal silver preparation reported to possess multiple biological activities [15]. The elemental form of zero-valent metallic silver particles contained in this product is coated with silver oxide, and the particle size is claimed by the manufacturer to range between 5–50 nm.

### Bacterial culture

The *P. aeruginosa* strain used in this study was sourced from our internal culture collection. This strain has been well characterized by us with respect to its antibiotic resistance, pigment production and certain other virulence traits. Its antibiogram generated through a disc-diffusion assay performed as per NCCLS guidelines revealed it to be resistant to 8 antibiotics (co-trimoxazole, augmentin, nitrofurantoin, ampicillin, chloramphenicol, clindamycin, cefixime, and vancomycin) belonging to 5 different classes (Fluoroquinolones, Beta lactams, Third generation cephalosporins, Macrolides, and Sulfonamides). Hence it can be described as a multidrug resistant (MDR) strain. As reported in our earlier publications [20, 21] involving this strain, it is a haemolytic strain capable of producing the quorum sensing (QS) regulated pigments (pyocyanin and pyoverdine), and also of biofilm formation.

As we also had *P. aeruginosa* PAO1 (MTCC 3541) strain in our lab, we could compare the MDR strain used in this study with the PAO1 in terms of their antibiotic resistance and virulence towards *Caenorhabditis elegans*. The strain used in our experiments was resistant to more classes of antibiotics, and it also displayed higher virulence towards the model host *Caenorhabditis elegans* (Table S1; Figure S1).

This bacterium was maintained on Pseudomonas agar (HiMedia). While culturing the bacterium for different assays, it was grown in Pseudomonas broth (magnesium chloride 1.4 g/L, potassium sulphate 10 g/L, peptic digest of animal tissue 20 g/L, pH 7.0 ± 0.2) supplemented with glycerol (3%v/v; HiMedia). Inoculum was added at 10%v/v, whose OD_625_ was adjusted between 0.08 and 0.10 to achieve equivalence to McFarland turbidity standard 0.5.

### Quantification of growth and quorum-regulated pigments

Effect of Silversol on bacterial growth and pigment formation was quantified through broth dilution assay. Following incubation (with intermittent shaking) of *P. aeruginosa* in Pseudomonas broth supplemented with or without Silversol for 21 ± 1 h at 35 °C, cell density was measured at 764 nm (Agilent Cary 60 UV-Vis). Pigments from the culture broth were extracted as described in [22] and [23]. One mL of culture broth was mixed in a 2:1 ratio with chloroform (Merck, Mumbai), followed by centrifugation (15,300 g) for 10 min. This resulted in formation of two immiscible layers. OD of the upper aqueous layer containing the yellow-green fluorescent pigment pyoverdine was measured at 405 nm. Pyoverdine Unit was calculated as OD_405_/OD_764_. The lower chloroform layer containing the blue pigment pyocyanin was mixed with 0.1 N HCL (20%v/v; Merck). This caused a change of colour from blue to pink. This was followed by centrifugation (15,300 g) for 10 min, and OD of upper layer acidic liquid containing pyocyanin was quantified at 520 nm. Pyocyanin Unit was calculated as OD_520_/OD_764_.

The lowest concentration of silver capable of inhibiting ≥ 80% growth was taken as minimum inhibitory concentration (MIC) [24]. From each tube showing absence of growth, 0.1 mL of broth was spread on Pseudomonas agar, and these agar plates were observed for appearance of growth over an incubation (at 35ºC) period of 72 h for determination of minimum bactericidal concentration (MBC). Extended incubation till 72 h was made in this case to be able to differentiate true bactericidal effect from any possible post-antibiotic effect [25, 26]. The concentration of silver which could inhibit appearance of growth on agar plates completely was taken as MBC.

### Haemolysis assay

Haemolytic activity is considered to be an important virulence trait of pathogens including *P. aeruginosa*, particularly under iron-limiting conditions. To investigate whether silver-exposure can have any impact on haemolytic potential of *P. aeruginosa*, we inoculated the silver-pre-treated cells on blood agar plate (HiMedia) and compared the haemolysis pattern with that of control.

OD_764_ of *P*. *aeruginosa* grown in Pseudomonas broth in presence or absence of Silversol was adjusted to 1.0, and 20 µL of this culture suspension was added onto the center of blood agar plate, followed by incubation at 35ºC for 24 h. Next day plates were observed for a zone of haemolysis surrounding the point of inoculation.

### Antibiotic susceptibility assay

Antibiogram of *P. aeruginosa*’s overnight grown culture in Pseudomonas broth in presence or absence of Silversol was generated through disc diffusion assay in accordance to Clinical and Laboratory Standards Institute (CLSI) guidelines [27]. Cells grown in Pseudomonas broth were separated through centrifugation (13,600 g) and washed with phosphate buffer (pH 7.0 ± 0.2) followed by centrifugation. The resulting cell pellet was used to prepare inoculum for subsequent disc diffusion assay by suspending the cells in normal saline and adjusting the OD_625_ between 0.08 and 0.10 to achieve an inoculum density equivalent to McFarland standard 0.5. One hundred µL of this inoculum was spread onto cation-adjusted Muller-Hinton agar (HiMedia) plates (Borosil; 150 mm) followed by placing the antibiotic discs (Icosa G-I MINUS; HiMedia, Mumbai) on the agar surface. Incubation at 35ºC was made for 18 ± 1 h, followed by observation and measurement of zone of inhibition.

### Biofilm assay

Biofilm formation is an important virulence trait, and hence effect of Silversol on biofilm forming ability of *P. aeruginosa*, as well as on pre-formed biofilm was investigated. A flow diagram depicting all four different biofilm assays is included in supplementary file (Figure S2). Biofilm quantification was achieved through crystal violet assay [28]. Biofilm viability was assessed through MTT assay [29].

For the crystal violet assay, the biofilm containing tubes (after discarding the inside liquid) were washed with phosphate buffer saline (PBS) in order to remove all non-adherent (planktonic) bacteria, and air-dried for 15 min. Then, each of the washed tubes was stained with 1.5 mL of 0.4% aqueous crystal violet (Central Drug House, Delhi) solution for 30 min. Afterwards, each tube was washed twice with 2 mL of sterile distilled water and immediately de-stained with 1.5 mL of 95% ethanol. After 45 min of de-staining, 1 mL of de-staining solution was transferred into separate tubes, and read at 580 nm (Agilent Cary 60 UV-Vis).

For the MTT assay, the biofilm-containing tubes (after discarding the inside liquid) were washed with PBS in order to remove all non-adherent (planktonic) bacteria, and air-dried for 15 min. Then 1.8 mL of minimal media (sucrose 15 g/L, K_2_HPO_4_ 5 g/L, NH_4_Cl 2 g/L, NaCl 1 g/L, MgSO_4_ 0.1 g/L, yeast extract 0.1 g/L, pH 7.4 ± 0.2) was added into each tube, followed by addition of 200 µL of 0.3% MTT [3-(4,5- Dimethylthiazol-2-yl)-2,5- iphenyltetrazolium Bromide; HiMedia]. Then after 2 h incubation at 35 °C, all liquid content was discarded, and the remaining purple formazan derivatives were dissolved in 2 mL of DMSO and measured at 540 nm.

### Exopolysaccharide (EPS) quantification

*P. aeruginosa* was grown in 100 mL flasks containing 20 mL of Pseudomonas broth. Incubation at 35ºC was made for 24 h with intermittent shaking. Following estimation of growth by measuring OD at 764 nm, culture broth was subjected to centrifugation (13,600 g for 10 min), and the supernatant was used for EPS quantification using the method described in [30] with some modification. Briefly, 40 mL of chilled acetone (Merck) was added to 20 mL of supernatant, and allowed to stand for 30 min. The EPS precipitated thus was separated by filtration through pre-weighed Whatman # 1 filter paper (Whatman International Ltd., England). Filter paper was dried at 60° C for 24 h, and weight of EPS on paper was calculated.

### Protein estimation

Extracellular protein present in bacterial culture (grown in presence or absence of silver for 21 ± 1 h at 35 °C) supernatant, and intracellular protein in the cell lysate was quantified through Folin-Lowry method [31, 32]. After measuring cell density, 1 mL of *P. aeruginosa* culture grown in Pseudomonas broth was centrifuged (13,600 g), and the resulting supernatant was used for extracellular protein estimation. The remaining cell pellet was subjected to lysis [33] for release of intracellular proteins. Briefly, the cell pellet was washed with phosphate buffer (pH 7.4), and centrifuged (13,600 g). Resulting pellet was resuspended in 1 mL of chilled lysis buffer (8.76 g/L NaCl, 10 mL of Triton X 100, 5/L g sodium deoxycholate, 1 g/L sodium dodecyl sulphate, and 6 g/L Tris HCl, in 990 mL of distilled water), and centrifuged (500 rpm) for 30 min at 4 °C for agitation purpose. This was followed by further centrifugation (16,000 g at 4 °C) for 20 min. Resulting cell lysate (supernatant) was used for protein estimation. Kanamycin (at IC_50_: 200 µg/mL), an aminoglycoside antibiotic known to inhibit bacterial protein synthesis [34], was used as a positive control.

### Nitrite estimation

Quantification of nitrite in bacterial culture was achieved through a colorimetric assay using modified Griess reagent [35]. A total of 250 µL of supernatant obtained from centrifugation (13,500 g for 10 min at 25 °C) of *P. aeruginosa* culture grown in presence or absence of silver (for 21 ± 1 h at 35 °C), was mixed with an equal volume of Griess reagent (1X concentration; Sigma-Aldrich) followed by 15 min incubation in dark at room temperature. Absorbance of the resulting colour was measured at 540 nm. This absorbance was plotted on the standard curve prepared using NaNO_2_ (0.43-65 µM) to calculate nitrite concentration. Sodium nitroprusside (Sigma Aldrich) was used as a positive control, as it is known to generate nitrosative stress in bacteria [36].

### Transcriptome analysis

To gain insights into the molecular mechanisms through which silver could inhibit bacterial growth and modulate various traits like QS, gene expression profile of *P. aeruginosa* challenged with sub-MIC of Silversol (1 ppm) was compared with that of control culture at the whole transcriptome level. Overall workflow of this whole transcriptome analysis (WTA) aimed at obtaining a holistic picture regarding mode of action of this formulation is presented in Figure S3.

#### RNA extraction and library preparation

RNA from bacterial cells was extracted by Trizol (Invitrogen Bioservices; 343,909) method, and dissolved in nuclease free water, followed by purity and concentration check, and quantifying RIN (RNA Integrity Number) values. Final libraries were quantified through Qubit 4.0 fluorometer (Thermofisher; Q33238). RIN values and acquired sizes of all libraries are reported in Table S2.

#### Genome annotation and functional analysis

Quality assessment of the raw fastq reads of the sample was performed using FastQC v.0.11.9 (default parameters) [37] and Fastp v.0.20.1 [38]. The *P. aeruginosa* genome (GCA_000006765.1_ASM676v1) was indexed using bowtie2-build [39] v2.4.2 (default parameters), and the processed reads were mapped to it using bowtie2 v2.4.2 parameters. The aligned reads from the individual samples were quantified using feature count v. 0.46. 1 [40] to obtain gene counts. These gene counts were used for differential expression estimation. The up- and down-regulated sequences were extracted from the *P. aeruginosa* coding file and subjected to blast2go [41] for annotation to extract the Gene Ontology (GO) terms. These GO terms were subjected to the wego [42] tool to obtain gene ontology bar plots.

All the raw sequence data has been submitted to Sequence Read Archive. Relevant accessions no. is SRX14392191 (https://www.ncbi.nlm.nih.gov/sra/SRX14392191).

### Network analysis

From the list of differentially expressed genes (DEG) in Silversol-exposed *P. aeruginosa*, those satisfying dual filter criteria of log fold-change ≥ 2 and FDR ≤ 0.05 were chosen for further network analysis. The list of such DEGs was fed into the STRING (v.11.5) database [43] to generate the PPI (Protein-Protein Interaction) network. Members of this PPI network were then arranged in decreasing order of ‘node degree’ (a measure of connectivity with other genes or proteins), and those above a specified threshold value were subjected to ranking by the cytoHubba plugin (v.3.9.1) [44] of Cytoscape [45]. As cytoHubba employs 12 different ranking methods, we considered the DEG being top-ranked by ≥ 6 different methods (i.e., 50% of the total ranking methods) for further investigation. These top-ranked shortlisted proteins were then subjected to local cluster analysis through STRING, and those that were part of multiple clusters were termed potential ‘hubs’ that can be investigated for additional validation of their targetability. The term ‘hub’ refers to a gene or protein that interacts with multiple other genes/proteins. The identified hubs were then subjected to co-occurrence analysis to see whether an antibacterial agent targeting them is likely to meet the requirement of selective toxicity (targeting the pathogen while causing no harm to the host). This sequence of analysis allowed us to end up with a limited number of proteins that satisfied multiple statistical and biological significance criteria simultaneously: (i) log fold-change ≥ 2; (ii) FDR ≤ 0.05; (iii) relatively higher node degree; (iv) top-ranking by at least 6 cytoHubba methods; (v) (preferably) member of more than 1 local network cluster; and (vi) high probability of the target being absent from the host.

### Polymerase chain reaction (RT-PCR)

Differential expression of the potential hubs identified through network analysis of DEG revealed from WTA was confirmed through PCR too. Primer designing for the selected genes was accomplished through Primer3 Plus [46]. Primer sequences thus obtained were confirmed for their binding withing the whole *P. aeruginosa* genome exclusively to the target gene sequence. Primer sequences for all the target genes are listed in Table 1. RNA extraction and quality check was done as described in preceding section. cDNA was obtained by using SuperScript™ VILO™ cDNA Synthesis Kit (Invitrogen Biosciences). PCR assay was conducted by using gene specific primers procured from Sigma-Aldrich. The gene PA3617 (recA) was kept as an endogenous control. The reaction mix used was FastStart Essential DNA Green Master mix (Roche; 06402712001). Real time PCR assay was performed on Quant studio 5 real time PCR machine (Thermo Fisher Scientific, USA). Temperature profile employed is provided in Table S3.

**Table 1:** Primer sequences for the target genes

Gene ID/ Name	Primers	Amplicon size (bp)
PA0524 (norB)	FP: 5’-CTACAACCCGGAAAACCTCA-3’	228
	RP: 5’-ACGCCGATCCAGAAGTAGTG-3’	
PA0521 (Cytochrome c subunit)	FP: 5’-CGAGCACGACACTTTCTTCA-3’	172
	RP: 5’-GACCATGTGCCAGTAGAGCA-3’	
PA3877 (narK1)	FP: 5’ -AAGACCGCATCCTTCATCAC-3’	238
	RP: 5’ -ACACCAGGAAGGTGAACAGC-3’	
PA3617 (recA) (selected as a negative control, not expected to express differently)	FP: 5’-CGTCAAGGTGGTGAAGAACA − 3’	231
	RP: 5’-TCCAGTACCGAACCGATTTC − 3’	

### Statistics

All results reported are means of three or more independent experiments, each performed in triplicate. Statistical significance was assessed through t-test performed using Microsoft Excel^®^, and data with p ≤ 0.05 was considered to be significant.

## Results and discussion

### Silver inhibits growth of *P. aeruginosa* and modulates QS-regulated pigment formation

**Fig. 1:**
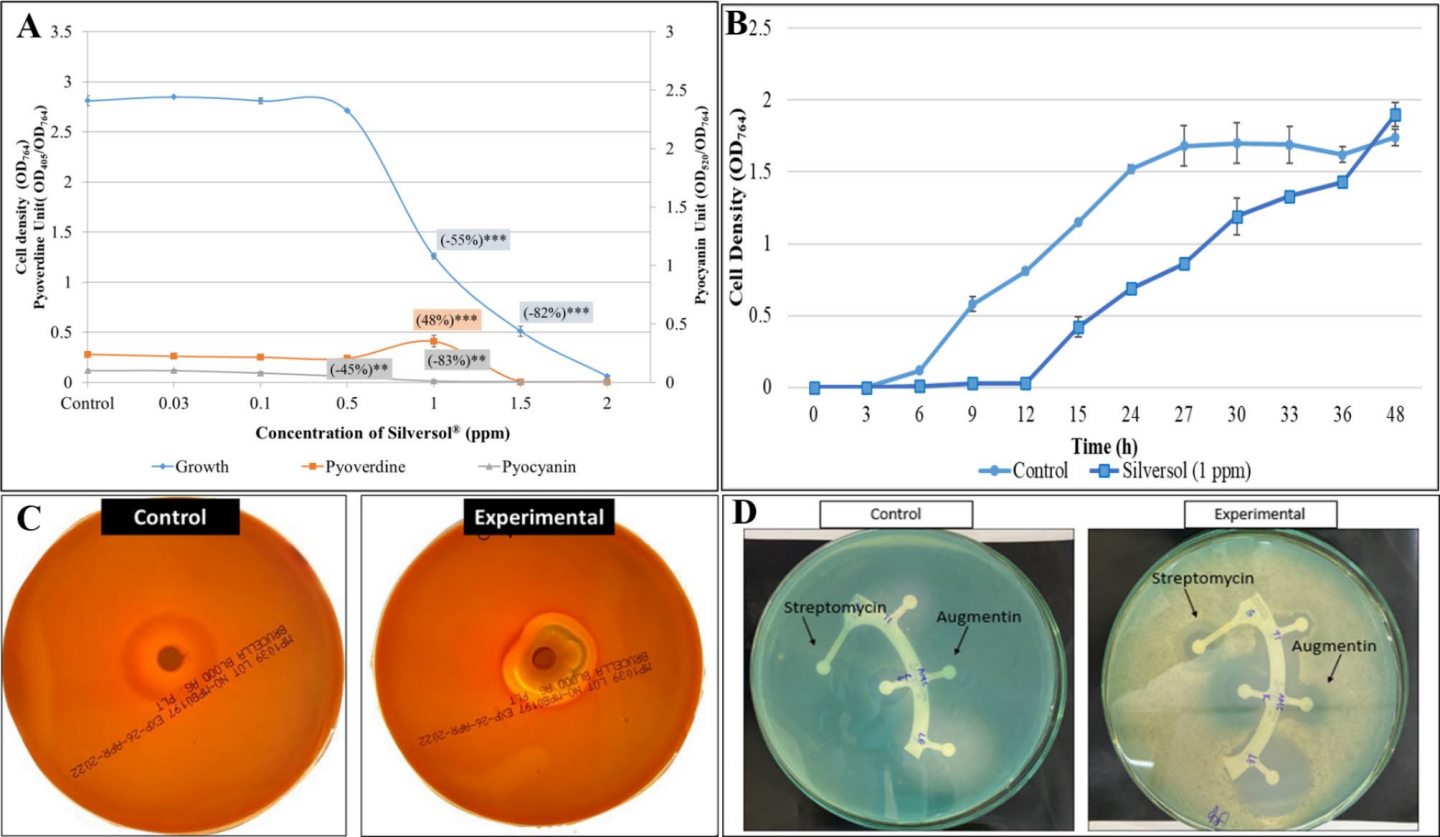
**(A)** Growth-inhibitory and quorum-modulatory effect of Silversol on *P. aeruginosa.* Bacterial growth was measured as OD_764_; OD of pyoverdine and pyocyanin was measured at 405 nm and at 520 nm respectively. Pyoverdine Unit and Pyocyanin Unit were calculated as the ratio OD_405_/OD_764_ and OD_520_/OD_764_(an indication of pigment production per unit of growth); Ofloxacin (0.3 µg/mL) inhibited growth by 76**%±0.1 with affecting pigment production. **p < 0.01, ***p < 0.001; minus sign (-) in parentheses indicate a decrease over control; **(B)** *P. aeruginosa* grows at slower growth rate in presence of sub-MIC of Silversol. Generation time of *P. aeruginosa* in presence and absence of Silversol was calculated to be 12.15 h and 11.17 h. For each time point shown in this growth curve, pigment quantification was also done. To avoid overcrowding of the graph, data pertaining to pigments is presented separately in Figure S5; **(C)** Silversol pre-treated *P. aeruginosa* exhibits enhanced haemolysis on blood agar plates. Silver-pretreated *P. aeruginosa* when inoculated onto blood agar plates, produced a clearer zone of haemolysis than its control counterpart receiving no previous exposure to silver; **(D)** Silversol pre-treatment enhances *P. aeruginosa*’s susceptibility to augmentin and streptomycin. Silver-pretreated cells seem to be altered in their response to antibiotics with respect to growth as well as QS-regulated pigmentation. On these plates of cation-adjusted Muller-Hinton agar, clear zones of inhibition can be seen surrounding the discs of augmentin and streptomycin in the experimental plate inoculated with silver-pre-treated cells, while such zones are absent (augmentin) or faint (streptomycin) from the control plate inoculated with *P. aeruginosa* having no previous silver-exposure

*P. aeruginosa* was challenged with different Silversol concentrations (0.03- 2 ppm). Bacterial growth and pyoverdine production remained unaffected till 0.5 ppm Silversol, while pyocyanin formation was inhibited in a dose-dependent fashion 0.5 ppm onward (Fig. 1A). Effect of Silversol on pyoverdine production appeared to follow an inverted U-shaped hormetic dose response curve [47] between the concentration range 0.5–1.5 ppm. Since Silversol inhibited pyocyanin formation at 0.5 ppm without affecting bacterial growth, this concentration can be said to be purely quorum modulatory. Since 1 ppm seemed to be the ~ IC_50_, and this concentration of Silversol also had a heavy effect on both quorum-regulated pigments, we chose this as the test concentration for further experiments. When growth curve of *P. aeruginosa* grown in presence of this sub-MIC concentration of Silversol (1 ppm) was compared with that grown in absence of Silversol over a period of 48-hour (Fig. 1B), it was observed that Silversol causes a delay in appearance of visible growth, and it allows cell density to reach only half of that of control culture till 27 h, however after that the difference between cell density of control and experimental culture starts getting narrow, and after 48 h of incubation, the cell density in both cultures gets at par statistically. This pattern of delayed onset of observed parameter in presence of Silversol, followed by recovery at par to the control culture in later phase of growth curve was observed in case of pigment production too [Figure S5 (A-B)], which is not surprising as production of both the pigments quantified here is regulated in a cell density-dependent manner in *P. aeruginosa* [48].

While Silversol seemed to inhibit pigment production completely 1.5 ppm onward, and caused complete visible inhibition of growth 2 ppm onward, the MBC was found to be somewhere between 16 and 20 ppm (Figure S4). When cells from experimental tubes were plated onto Pseudomonas agar (not containing any Silversol), only those coming from 1 ppm silver tubes gave pigmented growth, while those from 10–15 ppm silver tubes grew without pigmentation. Inoculum coming from the 20 ppm silver tubes failed to give rise to any growth on agar plates indicating that MBC has been reached.

### Silver appears to disturb iron homeostasis of *P. aeruginosa*

Silversol (1 ppm) enhanced pyoverdine production by almost 1.5-fold and inhibited pyocyanin heavily. Since pyoverdine is a siderophore [49], and pyocyanin also plays an important role in iron-sequestration [50, 51], we speculated that Silversol might be disturbing iron-homeostasis in *P. aeruginosa*. To have additional insight into this aspect, we inoculated blood agar plates with silver-pre-exposed *P. aeruginosa*, and these cells were found to be more haemolytic than their silver-not-exposed counterparts (Fig. 1C). Taking the effect of silver on pyoverdine production and haemolytic activity of the pathogen together, it can be said that silver-exposed cells seem to experience iron-limitation and to overcome this the cells are producing more siderophore and haemolysins. To further confirm the iron homeostasis-disturbing activity of Silversol, we grew *P. aeruginosa* in Pseudomonas broth supplemented with Silversol as well as different concentrations of ferrous sulphate (Merck) to check whether sufficient iron supply can protect *P. aeruginosa* from Silversol’s antibacterial effect. Indeed, *P. aeruginosa* was able to remain resistant to Silversol’s growth-inhibitory effect in presence of FeSO_4,_ and it also produced lesser pyoverdine than when growing in Pseudomonas broth with or without Silversol [Figure S5(C)]. It can be said that in presence of additional iron in growth media, the bacterium is not forced to make extra efforts for iron scavenging in terms of pyoverdine production, and hence Silversol’s negative effect on bacterial iron-homeostasis is confirmed, and this effect may be much more detrimental in iron-limiting conditions inside a host. Most microbes need iron at a concentration of 10^− 6^ M for their growth and reproduction, while only minute quantities (~ 10^–15^ M) of free iron are available in the human body [52].

### Silver-treated *P. aeruginosa* displays higher susceptibility to augmentin and streptomycin

To investigate whether silver exposure influences antibiotic susceptibility of *P. aeruginosa*, we grew *P. aeruginosa* in presence of 1 ppm Silversol, and the antibiogram of the resulting cells (after washing with phosphate buffer) was compared to that of control cells. Silver-pre-exposed cells displayed a phenotype change from ‘resistant’ to ‘sensitive’ against augmentin; and a notable increase (38%) in susceptibility to streptomycin (Table 2; Fig. 1D). Such resistance-modifying activity of the silver becomes quite important given *P. aeruginosa* is known rarely to be susceptible to augmentin [53], and augmentin resistance is quite prevalent among its clinical isolates [54]. Streptomycin-resistance was reported by [55] to be among the most common resistance patterns in *P. aeruginosa* clinical isolated from Mexico.

### Silversol could partially eradicate pre-formed *P. aeruginosa* biofilm

**Fig. 2:**
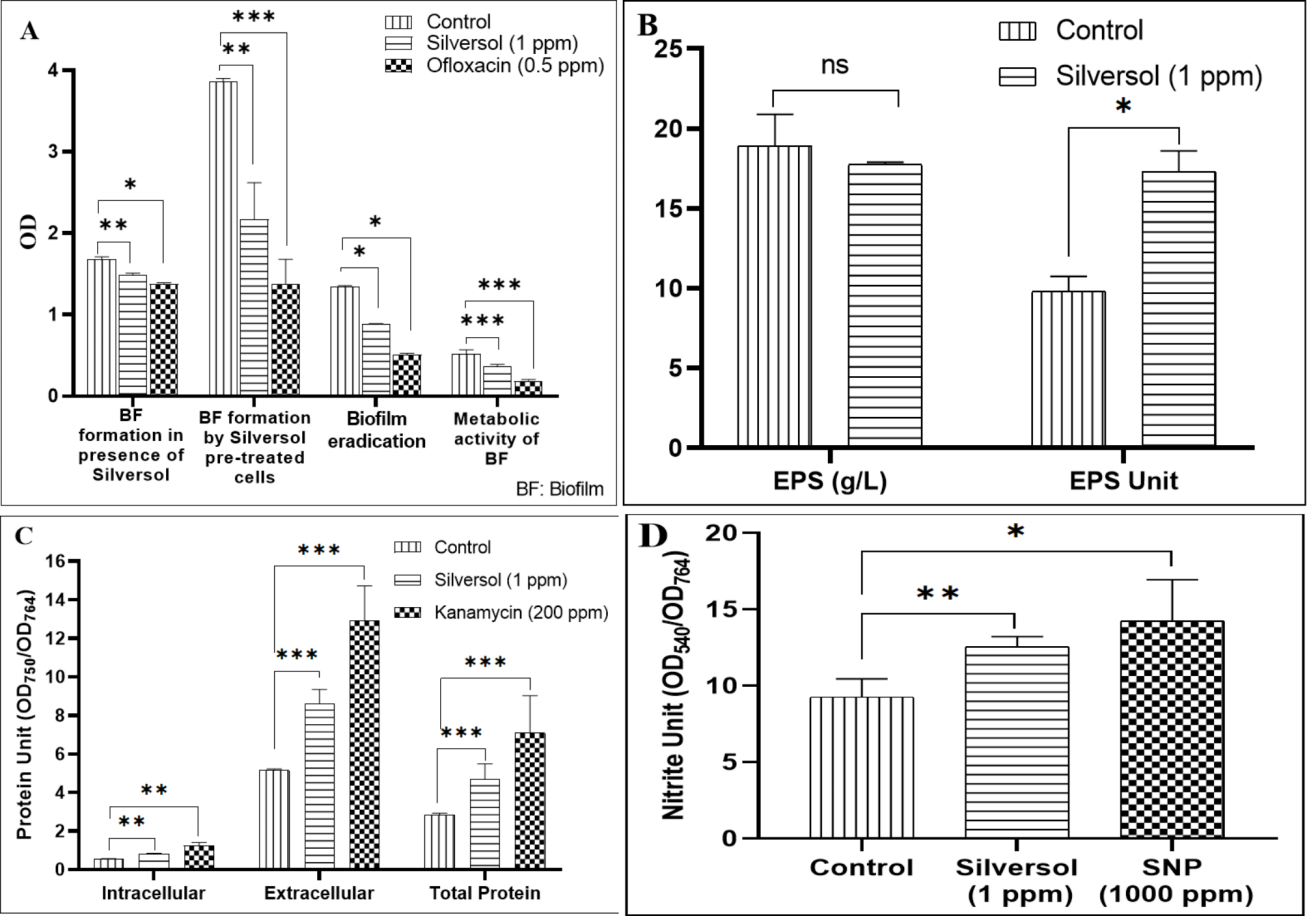
Silver modulates biofilm formation, exopolysaccharide synthesis, protein synthesis, and nitrogen metabolism in *P. aeruginosa*. **(A)** Silversol could partially inhibit *P. aeruginosa* biofilm formation, and eradicate the pre-formed biofilm. Crystal violet assay was performed to quantify biofilm formation, and eradication. Cell viability in the biofilm was estimated through MTT assay; (**B)** Silversol enhances EPS synthesis in *P. aeruginosa*. Though there are lesser number of cells in the silver-supplemented media, they synthesized EPS in amount equal to their silver-unexposed counterparts. EPS Unit was calculated as Cell Density (OD_764_): EPS (g/L) ratio; (**C)** *P. aeruginosa* grown in presence of Silversol registered higher protein synthesis. Intracellular and extracellular protein concentrations in *P. aeruginosa* grown in presence of Silversol at sub-MIC level were significantly higher as compared to its silver-non-exposed counterpart. Kanamycin employed as a positive control at its sub-MIC level also generated similar response from bacterial culture. Protein Unit (i.e., Protein concentration: Cell density ratio) was calculated to nullify any effect of cell density on protein production; (**D)** Silversol-treated *P. aeruginosa* culture has higher extracellular accumulation of nitrite. Silversol caused nitrite concentration in *P. aeruginosa* culture supernatant to rise when compared to control. Sodium nitroprusside (SNP) used as a positive control also caused higher nitrite build up in *P. aeruginosa* culture. Nitrite Unit (i.e., Nitrite concentration: Cell density ratio) was calculated to nullify any effect of cell density on nitrite production. *p < 0.05, **p < 0.01, ***p < 0.001, ns: non-significant

While Silversol inhibited biofilm formation in *P. aeruginosa* partially (19%), it could also eradicate the pre-formed biofilm (by 34%) and reduce biofilm viability by 30%. Inhibitory effect of Silversol on *P. aeruginosa* biofilms seemed independent from its growth-inhibitory effect, since Silversol-pre-treated cells subsequently allowed to form biofilm in test-tubes filled with Silversol-free growth media, could form 43% lesser biofilm (Fig. 2A). Since bacterial biofilms are known for displaying higher antibiotic resistance than their planktonic counterparts [56], biofilm-eradicating agents can help restoring the antibiotic susceptibility of bacterial cells by freeing them of the biofilm matrix.

Since exopolysaccharides (EPS) constitute an important component of bacterial biofilm matrix, we quantified EPS in control vs. silver-treated *P. aerugin*osa culture. Though the absolute EPS in experimental and control cultures was at par, nullifying it against reduced cell density in silver-exposed culture, EPS production in experimental culture was found to be enhanced by 76.34% (Fig. 2B). Higher EPS synthesis can be considered as an indication of envelope stress [57] in the silver-exposed bacteria. When faced with a variety of stress conditions, *P. aeruginosa* is known to respond by escalating its EPS synthesis as a survival strategy, since EPS serves as a protective matrix [58, 59], whose production is upregulated during nutrient limitation and oxidative stress [60, 61]. This study found the sub-MIC level of Silversol to disturb QS-regulated traits in *P. aeruginosa* such as pigmentation [Fig. 1A and Figure S5(A)-(B)] and biofilm formation (Fig. 2A), and the quorum sensing system in *P. aeruginosa* is known to influence EPS production too [62]. Studying the correlation between EPS production and stress-response in *P. aeruginosa* can provide new insights into its survival mechanisms and pathogenicity.

### *P. aeruginosa* seemed to upregulate its protein synthesis in presence of silver

*P. aeruginosa* exposed to sub-MIC of Silversol was found to have higher intracellular as well as extracellular protein concentration than its silver-non-exposed counterpart (Fig. 2C). Bacterial response to the known inhibitor of protein synthesis (Kanamycin) was also similar. It may be said that bacteria responds to the inhibitory effect of such antimicrobials exerting their action through suppressing protein synthesis, by upregulating its protein sysnthesis and/or secretion machinery to compensate the inhibitory effect of the antimicrobial agents [63]. Such upregulation of protein synthesis might have stemmed from the translational reprogramming in the stressed cells [64], as they are facing the stress of the antibacterial activity of the silver.

**Table 2:** Silver pre-treatment modulates bacterial susceptibility to some antibiotics

Antibiotic	Concentration (µg/disc)	Zone of inhibition		% Difference
		**Control** (Mean ± SD)	**Experimental** (Mean ± SD)	
Imipenem	10	29 ± 2.82	30.5 ± 0.70	Non-significant
Ciprofloxacin	5	40 ± 4.24	39.5 ± 6.36	
Tobramycin	10	29.5 ± 0.70	29 ± 2.82	
Moxifloxacin	5	35.5 ± 0.70	33 ± 2.82	
Ofloxacin	5	34 ± 0	34.5 ± 3.53	
Sparfloxacin	5	33 ± 1.4	31 ± 5.65	
Levofloxacin	5	38 ± 1.41	36.5 ± 2.12	
Norfloxacin	10	34.5 ± 0.70	33.5 ± 2.12	
Co-Trimoxazole	25	**R**	**R**	
Nalidixic acid	30	15 ± 10.6	15.5 ± 0.70	
**Augmentin**	**30**	**R**	**10.5 ± 0.70**	**Resistance reversal**
Gatifloxacin	5	34.65 ± 0.49	34 ± 1.41	Non-significant
Amikacin	30	22 ± 1.41	21.5 ± 0.70	
**Streptomycin**	**25**	**13 ± 0**	**18 ± 1.41**	38.46*
Ceftriaxone	30	25.5 ± 2.12	23.5 ± 3.53	Non-significant
Cefpodoxime	10	25 ± 0	25.5 ± 0.70	
Colistin	10	21.7 ± 0.7	22 ± 0.6	
Kanamycin	30	18.2 ± 1.06	21 ± 1.41	
Gentamicin	10	21.5 ± 0.7	2.05 ± 0.7	
Ticarcillin	75	26.7 ± 1.06	25.5 ± 0.7	

Antibiotic susceptibility profile of the bacterium was generated using the antibiotic discs- Icosa G-I Minus (HiMedia, Mumbai), through disc diffusion assay performed as per NCCLS guidelines. The zones of inhibition were measured and the interpretation (S - sensitive, I - intermediate, R - resistant) was drawn as per zone size interpretative chart provided by the manufacturer. *p < 0.05

### Silver induces nitrosative stress in *P. aeruginosa*

Since denitrification pathway is an important metabolic pathway in *P. aeruginosa*, and enzymes involved in detoxification of reactive nitrogen species are proposed to be potential anti-pathogenic targets [65, 66], we quantified one of the intermediates of denitrification pathway, nitrite (NO_2_^-^), in silver-exposed *P. aeruginosa*. Culture supernatant of the latter was found to possess 37% higher nitrite (Fig. 2D) than that grown in absence of silver. Higher nitrite concentrations can have multiple effects on *P. aeruginosa* physiology and virulence. It can exert its toxicity by disrupting electron transport chain, and can also impair bacterial virulence, besides modulating susceptibility to various antibiotics. Nitrite can react with other reactive nitrogen species (RNS) to form highly reactive intermediates, such as peroxynitrite (ONOO^-^), which can damage cellular components, including proteins, lipids, and DNA [67]. Nitrite can interfere with the electron transport chain in *P. aeruginosa*, leading to a decrease in ATP production and compromised energy metabolism [68, 69]. This disruption can negatively impact various cellular processes and growth. Nitrite can also inhibit the production of virulence factors such as pyocyanin, elastase, and siderophores [70]. Nitrite build-up can negtively impact bacterial physiology by trigerring the overexpression of efflux pumps and altering membrane permeability [71].

### Differential gene expression in silver-treated *P. aeruginosa*

Silversol-exposed *P. aeruginosa* when subjected to whole transcriptome analysis, it was found to express a total of 26 genes (0.48% of total genome) differently (log fold-change ≥ 2 and FDR ≤ 0.05). List of differentially expressed genes (DEG) is provided in Table 3, and corresponding heat map (Figure S6) and volcano plot (Figure S7) can be seen in supplementary file. When we looked for functions of the DEG in appropriate databases like KEGG, PDB, or UniProt, all the 6 downregulated genes turned out to be hypothetical proteins. Amongst the upregulated genes in silver-exposed *P. aeruginosa* culture, 13 genes were associated with nitrogen metabolism, 2 were associated with heme biosynthesis or acquisition, three were hypothetical proteins, 1 was pyoverdine regulator and 1 was a protein folding catalyst. Function-wise categorisation of all DEG is presented in Figure S8.

Empirically the transcriptomic profile indicated Silversol to disturb iron homeostasis and nitrogen metabolism in *P. aeruginosa*. The hypothesis of disturbance of iron homeostasis corroborates well with results of our in vitro experiments for assessing haemolytic potential (Fig. 1C) and pyoverdine [Fig. 1A and Figure S5 (C)] production. It seems that in presence of Silversol, the bacteria are facing iron-limitation and is forced to upregulate the genes coding for heme synthesis and siderophore production to maintain iron homeostasis. This observation is important in light of the fact that iron is an essential micronutrient for bacteria, and since the concentration of free iron in human serum is quite low [72], pathogens have to develop iron-scavenging strategies for a successful in-host survival. Differential expression of multiple genes associated with nitrogen metabolism also corroborates well with increased nitrite concentration in silver-treated bacterial culture supernatant (Fig. 1D).

**Fig. 3:**
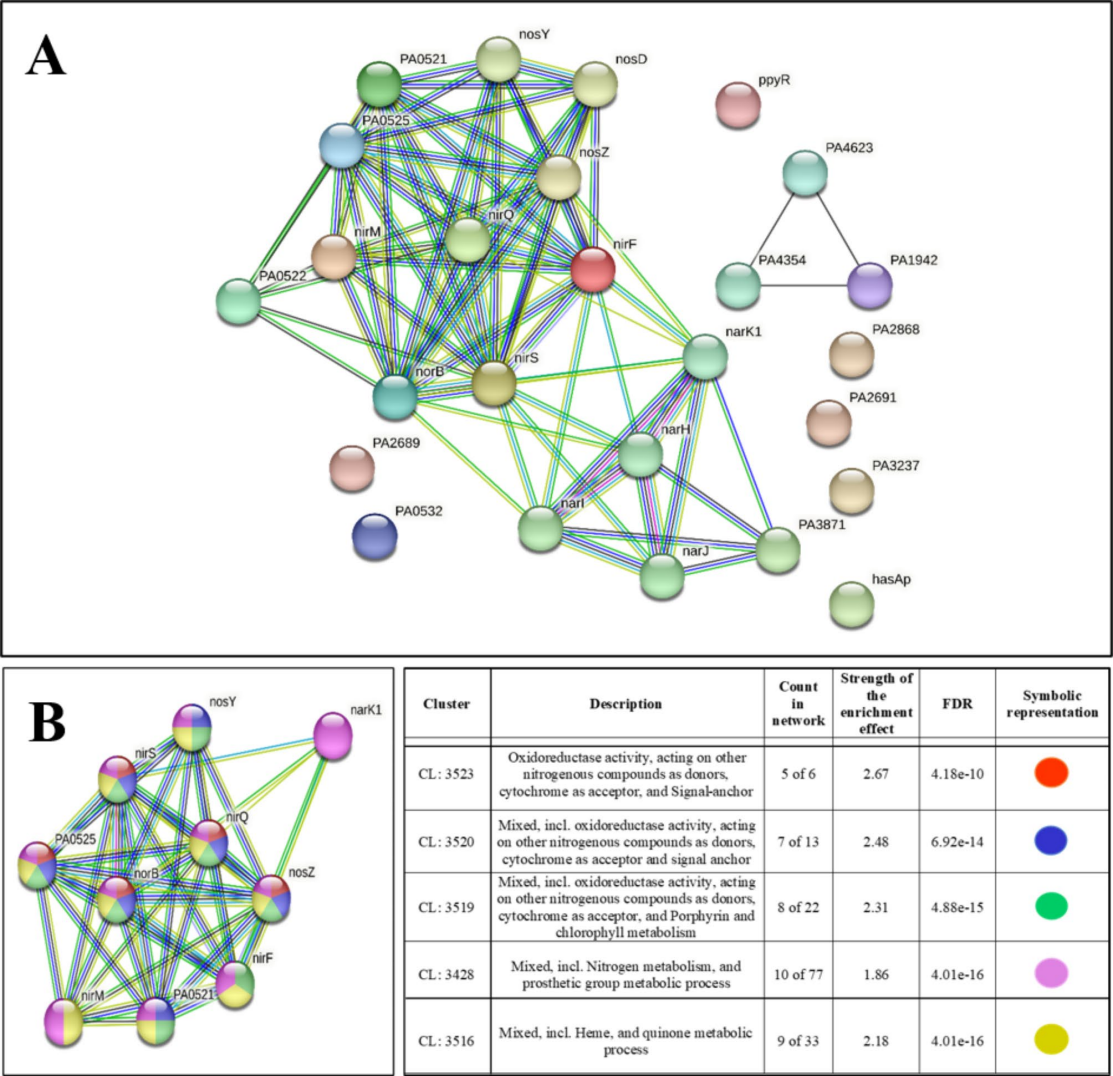
**(A)** PPI network of DEGs in Silversol-exposed *P. aeruginosa;***(B)** PPI network of top-ranked genes revealed through cytoHubba among DEG in Silversol*-*exposed *P. aeruginosa*

To have a holistic idea of the antibacterial mechanism of Silversol, we subjected all the DEG to network analysis through STRING. The resulting protein-protein interaction (PPI) (Fig. 3A) network had 26 nodes connected through 74 edges with an average node degree of 5.69. Since the number of edges (74) in this PPI network is almost 25-fold higher than expected (03) with a PPI enrichment p–value < 1.0e-16, this network can be said to possess significantly more interactions among the member proteins than what can be expected for a random set of proteins having identical sample size and degree distribution. Such an enrichment is suggestive of the member proteins being at least partially biologically connected.

**Table 3:** List of DEGs in Silversol exposed *P. aeruginosa* satisfying the dual criteria of log fold-change ≥ 2 and FDR ≤ 0.05

Sr. No.	Gene ID	Gene Symbol	Codes for	Log FC	FDR	Up- or down-regulation	Node degree
1	PA0522	NA	hypothetical protein	4.9	5.87E-06	↑	6
2	PA2691	NA	conserved hypothetical protein	4.2	5.10284E-05	↑	0
3	PA2689	NA	hypothetical protein	4.02	0.0002	↑	0
4	PA0521	NA	probable cytochrome c oxidase subunit	3.91	0.0002	↑	10
5	PA4623	NA	hypothetical protein	3.5	0.001	↓	2
6	PA3237	NA	hypothetical protein	3.47	0.001	↓	0
7	PA0524	norB	nitric-oxide reductase subunit B	3.41	0.001	↑	13
8	PA3877	narK1	nitrite extrusion protein 1	3.27	0.003	↑	9
9	PA3872	narI	respiratory nitrate reductase gamma chain	3.21	0.01	↑	7
10	PA2868	NA	hypothetical protein	3.16	0.01	↓	0
11	PA3871	NA	probable peptidyl-prolyl cis-trans isomerase, PpiC-type	3.01	0.02	↑	4
12	PA0532	NA	hypothetical protein	2.99	0.01	↓	0
13	PA3395	nosY	NosY protein	2.98	0.02	↑	8
14	PA0520	nirQ	regulatory protein NirQ	2.9	0.02	↑	11
15	PA3873	narJ	respiratory nitrate reductase delta chain	2.89	0.04	↑	5
16	PA0516	nirF	heme d1 biosynthesis protein NirF	2.84	0.02	↑	12
17	PA2663	ppyR	psl and pyoverdine operon regulator, PpyR	2.82	0.05	↑	0
18	PA0518	nirM	cytochrome c-551 precursor	2.82	0.03	↑	8
19	PA0519	nirS	nitrite reductase precursor	2.74	0.03	↑	14
20	PA0525	NA	probable dinitrification protein NorD	2.73	0.03	↑	10
21	PA3392	nosZ	nitrous-oxide reductase precursor	2.72	0.03	↑	10
22	PA4354	NA	conserved hypothetical protein	2.7	0.04	↓	2
23	PA3393	nosD	NosD protein	2.6	0.05	↑	8
24	PA3407	hasAp	heme acquisition protein HasAp	2.59	0.05	↑	0
25	PA3874	narH	respiratory nitrate reductase beta chain	2.58	0.05	↑	7
26	PA1942	NA	hypothetical protein	2.57	0.05	↓	2

Genes are arranged in decreasing order of Fold Change. Databases consulted for gene functions were: NCBI gene database (https://www.ncbi.nlm.nih.gov/nuccore/NC_002516); KEGG (Kyoto Encyclopaedia of Genes and Genomes: https://www.genome.jp/kegg/); Uniprot (https://www.uniprot.org/). NA: not available

When we arranged all the 26 nodes in decreasing order of node degree, 19 nodes were found to have a non-zero score, and we selected top 16 genes with a node degree ≥ 4 for further ranking by different cytoHubba methods. Then we looked for genes which appeared among top ranked candidates by ≥ 6 cytoHubba methods. This allowed us to shortlist ten genes (Table 4) which were ranked among top-10 by ≥ 9 cytoHubba methods to be taken for further cluster analysis. Interaction map of these 10 important genes (Fig. 3B) showed them to be networked with the average node degree score of 8. Number of edges possessed by this network was 40 as against expected 0 for any such random set of proteins. These 10 genes were found to be distributed among five different local network clusters. Strength score for each of these clusters was > 1.86. While five of the proteins (norB, PA0525, nirS, nirQ, and nosZ) were members of all the five clusters, two proteins (nosY and PA0521) were part of four different clusters. Of the remaining three, nirF was part of three different clusters, while nirM and narK1 were respectively members of two and a single cluster. The multi-cluster proteins identified here can be said as not only the most important targets of Silversol, but also hub proteins with high network centrality and potential targets for novel anti-Pseudomonas drug discovery programmes.

**Fig. 4:**
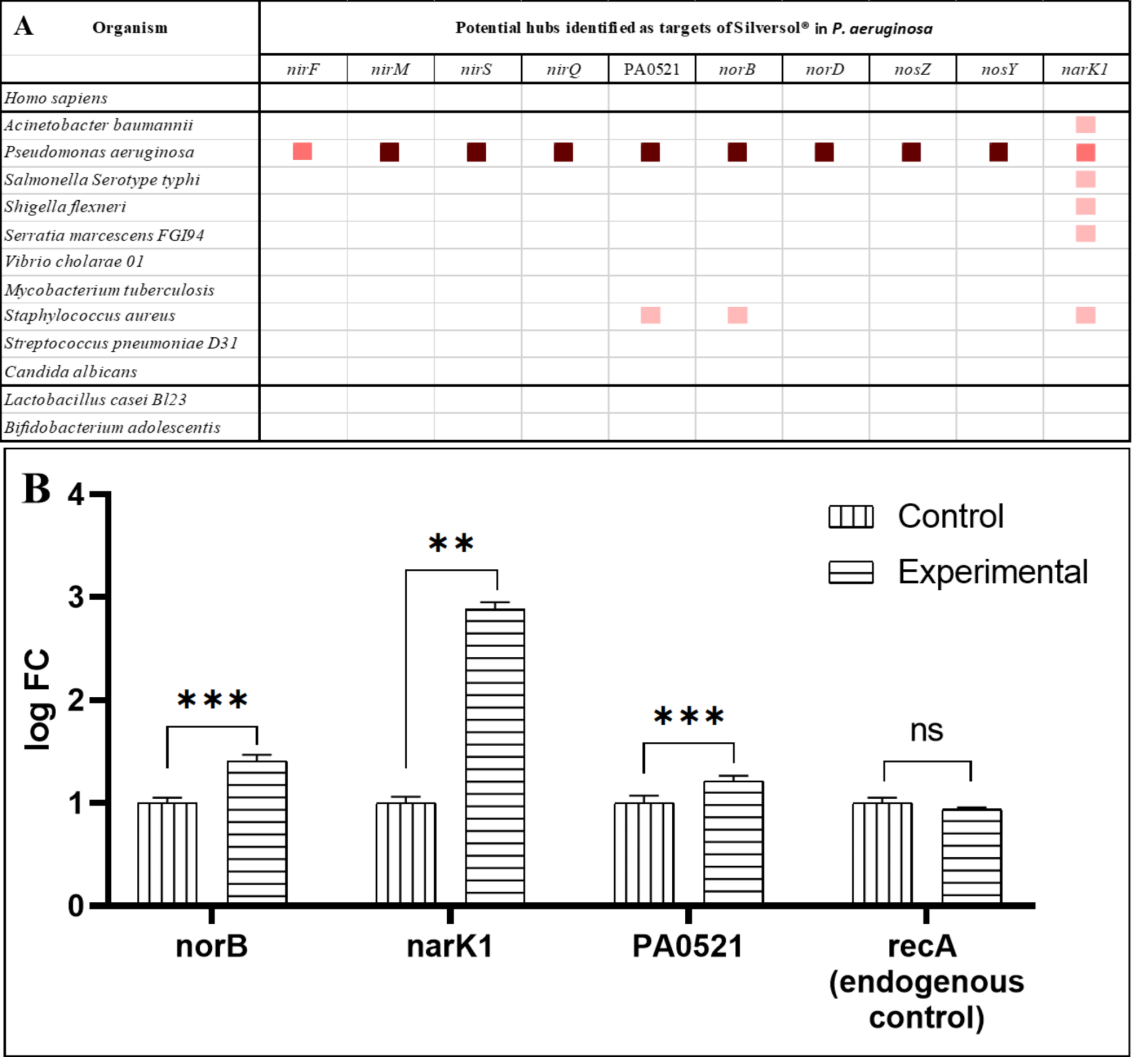
**(A)** Cooccurrence analysis of genes coding for potential targets in *P. aeruginosa*; **(B)** Confirmation of differential expression of selected genes in Silversol-treated *P. aeruginosa* through RT-PCR. ***p < 0.001; ** p < 0.01

We also conducted a gene cooccurrence pattern analysis of gene families across genomes (through STRING) with respect to the potential hubs identified by us (Fig. 4A). None of the 10 potential targets was shown to be present in humans, hence any anti-pathogenic drugs capable of causing dysregulation of these genes are least likely to toxic to humans. This corroborates well with reports showing Silversol to be safe for human consumption [15]. Three of the Silversol^’^s target genes in *P. aeruginosa* were also shown to be present in *Staphylococcus aureus*. However, except narK1, no other target proteins were shown to be present in other important pathogens. Since Silversol is already known to be active against a wide spectrum of gram-positive and gram-negative bacteria [15], it may be believed to be acting against different organisms through different mechanisms.

One of the side-effect with conventional antibiotics is that they fail to differentiate between the ‘good’ (symbionts in human microbiome) and ‘bad’ (pathogens) bacteria, and hence their therapeutic use may lead to gut dysbiosis. An ideal antimicrobial agent is expected to target pathogens exclusively without causing gut dysbiosis. In this respect, a target in pathogenic bacteria absent from symbionts of human microbiome will be most suitable candidate for antibiotic discovery programmes. To have some insight on this front with respect to the targets identified by us, we run a gene cooccurrence analysis with two representative ‘good’ bacteria too, reported to be part of healthy human microbiome, and the said targets were not shown to be present in them. This corroborates with the selective inaction of Silversol on probiotic strains of *Lactobacillus acidophilus* and *Bifidobacterium longum* documented in internal reports of Viridis Biopharma (Selective inaction of ASAP on probiotics. Unpublished raw data, 2004).

### Target validation through RT-PCR

Based on the network analysis of transcriptome of silver-treated *P. aeruginosa*, we selected following three genes for further validation through RT-PCR: nor B (identified as member of all the 5 clusters in network analysis), PA0521 (identified as member of 4 different clusters in network analysis), and narK1 (shown by gene cooccurrence analysis to be present across multiple pathogenic genera); wherein they were found to be up-regulated in silver-treated *P. aeruginosa* by 2.65-fold, 2.32-fold, and 7.37-fold respectively (Fig. 4B).

**Table 4:** Top ten cytoHubba-ranked genes from among the top-16 with respect to node degree

No.	Gene ID	Gene Name	Number of methods ranking this protein among top ten	Names of 12 ranking methods of CytoHubba and rank score provided by them											
				Degree	MNC	DMNC	MCC	Bottleneck	EcCentricity	Closeness	Radiality	Betweenness	Stress	CC	EPC
1	PA0520	nirQ	12	11	11	0.729	46,200	1	0.5	13	3	5.788	36	0.781	6.331
2	PA3392	nosZ	12	10	10	0.758	45,480	3	0.5	12.5	2.93	3.404	24	0.844	6.255
3	PA0519	nirS	10	14	14	-	46,344	7	0.5	14.5	3.2	34.704	124	-	6.928
4	PA0524	norB	10	13	13	0.638	46,320	-	0.5	14	3.13	18.621	92	-	6.827
5	PA0516	nirF	10	12	12	0.658	45,600	-	0.5	13.5	3.06	13.738	72	-	6.663
6	PA0521	Cyto.c	10	10	10	0.778	46,080	-	-	12.33	2.86	2.038	12	0.866	6.084
7	PA0525	norD	9	10	10	0.778	46,080	-	-	12.33	2.86	2.038	-	0.866	6.064
8	PA3877	narK1	9	9	9	-	-	2	0.5	12	2.86	15.750	82	-	5.714
9	PA0518	nirM	9	8	8	0.758	5760	1	0.33	11.33	2.73	-	-	0.928	-
10	PA3395	nosY	9	8	8	0.816	40,320	1	-	11.33	2.73	-	-	1	5.642

‘-’indicates that the given method did not rank that particular gene among top ten

## Conclusion

**Fig. 5:**
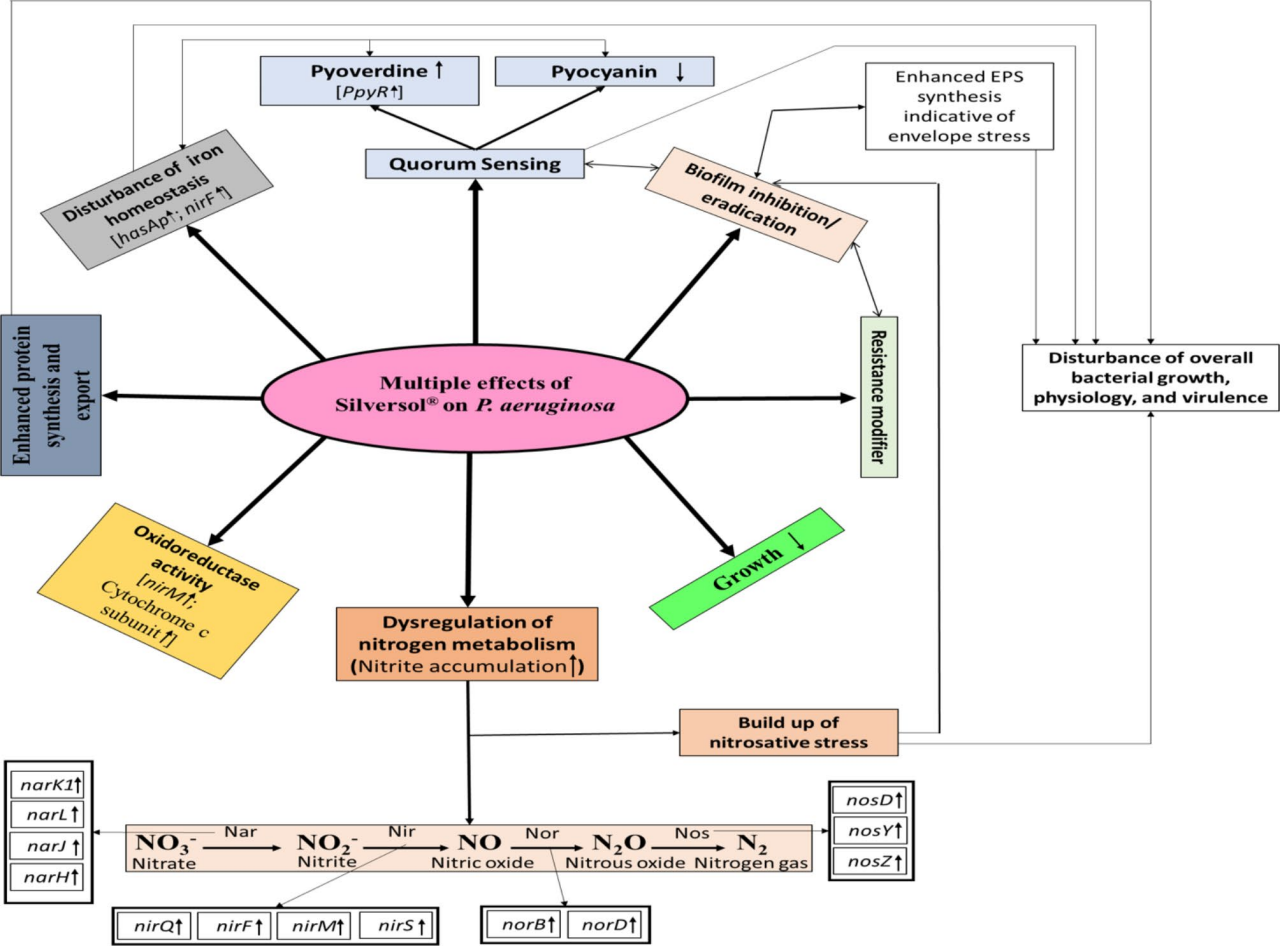
Overall schematic of multiple effects of Silversol on *Pseudomonas aeruginosa.* Various physiological and virulence traits of *P. aeruginosa* affected under the influence of sub-lethal concentration of Silversol are depicted. Up (↑) or down (↓) regulation of the genes relevant to those traits is also indicated. EPS: exopolysaccharide

Silver has long been known for its antimicrobial activity. Though literature contains reports describing a variety of modes of antibacterial action of silver and its nanoparticles, our knowledge on this front is far from complete. Antibacterial mechanisms of silver previously described by various researchers include generation of oxidative stress [73–75], generating nitrosative stress [75], disruption of membrane integrity [76], protein denaturation [77, 78], inhibition of enzymatic activity [79, 80], interference with quorum sensing [81, 82], inducing DNA damage [74] and interfering with its replication and transcription [83], inhibition of efflux pumps [81], etc. The present study investigated effect of sub-MIC level of colloidal silver on *P. aeruginosa*’s growth, QS-regulated pigmentation, biofilm, protein synthesis, nitrogen metabolism, EPS synthesis, haemolytic activity and siderophore production, antibiotic susceptibility, and gene expression at the whole transcriptome level. A schematic summary of Silversol’s multiple effects on *P. aeruginosa* is presented in Fig. 5. Disruption of iron homeostasis and generation of nitrosative stress seemed to be the major mechanisms of anti-*P. aeruginosa* activity of silver in this study. Differential expression of three important genes involved in denitrification pathway in silver-exposed *P. aeruginosa* was confirmed through RT-PCR too. Hub proteins identified in this study as major targets of silver in *P. aeruginosa* warrants further investigation with respect to validating their targetability e.g., by confirming defective growth of mutant strains of *P. aeruginosa* bearing deletion of one or more of the identified hub genes. Such validated targets can prove vital to various antibiotic discovery efforts globally. Though the gene expression profile of the bacterium under influence of sub-lethal concentrations of silver may vary from that under influence of its lethal concentrations, this study provides useful insights into antibacterial mechanism of silver and identification of its potential targets in *P. aeruginosa.*

### Electronic supplementary material

Below is the link to the electronic supplementary material.

Supplementary Material 1

## Data Availability

All the data has been provided within main manuscript or supplementary files. All the raw sequence data has been submitted to Sequence Read Archive. Relevant accessions no. is SRX14392191 (https://www.ncbi.nlm.nih.gov/sra/SRX14392191).
