# Supplementary material for: Sub-lethal concentration of a colloidal nanosilver formulation (Silversol®) triggers dysregulation of iron homeostasis and nitrogen metabolism in multidrug resistant Pseudomonas aeruginosa
Source: BMC Microbiol. 2023 Oct 23;23:303. doi: 10.1186/s12866-023-03062-x (PMC10591374; doi:10.1186/s12866-023-03062-x)
Supplement: Supplementary file 1 — Supplementary Material 1 [file 12866_2023_3062_MOESM1_ESM.docx]

**Supplementary file**

**Sub-lethal concentration of a colloidal nanosilver formulation (Silversol^®^) triggers dysregulation of iron homeostasis and nitrogen metabolism in multidrug resistant *Pseudomonas aeruginosa***

Gemini Gajera^1^, Nidhi Thakkar^1^, Chhaya Godse^2^, Anselm DeSouza^2^, Dilip Mehta^2^, Vijay Kothari^1*^

^1^Institute of Science, Nirma University, Ahmedabad- 382481, India

^2^Viridis BioPharma Pvt Ltd, Mumbai, India

^*^Correspondence: vijay.kothari@nirmauni.ac.in

**Figure S1. MDR strain of *P. aeruginosa* used in our study could kill the host worms faster than the PAO1 strain**

When worms in liquid media were challenged with either strain of *P. aeruginosa,* it took 18 h for the MDR strain to kill cent percent of the worm population as against 22 h required by PAO1 to achieve same effect. At the 12-hour and 18-hour end-points, MDR could kill 40%±1*** and 40%±5.7*** more worms than PAO1. ***p<0.001

**Table S1. Comparison of antibiograms of *Pseudomonas aeruginosa* (MDR strain used in this study) and PAO1 generated through Kirby-Bauer Disc Diffusion assay**

| **Sr. No.** | **Antibiotic** | **Concentration (μg per disc)** | ***Pseudomonas aeruginosa***  **(MDR)** | ***Pseudomonas aeruginosa* PAO1** |
| --- | --- | --- | --- | --- |
| 1 | Ceftriaxone | 30 | S | S |
| 2 | Colistin | 10 | S | S |
| 3 | Ciprofloxacin | 5 | S | S |
| 4 | Co-Trimoxazole | 25 | R | R |
| 5 | Imipenem | 10 | S | S |
| 6 | Ticarcillin | 75 | S | S |
| 7 | Streptomycin | 25 | S | S |
| 8 | Sparfloxacin | 5 | S | S |
| 9 | Cefpodoxime | 10 | S | S |
| 10 | Nalidixic Acid | 30 | S | S |
| 11 | Moxifloxacin | 5 | S | S |
| 12 | Gentamicin | 10 | S | S |
| 13 | Gatifloxacin | 5 | S | S |
| 14 | Ofloxacin | 5 | S | S |
| 15 | Tobramycin | 10 | S | S |
| 16 | Norfloxacin | 10 | S | S |
| 17 | Amikacin | 30 | S | S |
| 18 | Levofloxacin | 5 | S | S |
| 19 | Augmentin | 30 | R | R |
| 20 | Kanamycin | 30 | S | S |
| 21 | Vancomycin | 30 | R | R |
| 22 | Cefixime | 5 | R | R |
| 23 | Tetracycline | 30 | I | S |
| 24 | Rifampicin | 5 | I | R |
| 25 | Clindamycin | 2 | R | R |
| 26 | Chloramphenicol | 30 | R | S |
| 27 | Cefepime | 30 | S | S |
| 28 | Doxycycline Hydrochloride | 30 | I | I |
| 29 | Cefotaxime | 30 | S | S |
| 30 | Nitrofurantoin | 300 | R | R |
| 31 | Ampicillin | 10 | R | R |

Antibiotic susceptibility profile of the organisms was generated using the antibiotic discs- Icosa G-I Minus (HiMedia, Mumbai) through disc diffusion assay on cation-adjusted Mueller-Hinton agar (HiMedia) as per National Committee for Clinical Laboratory Standards (NCCLS) guidelines (https://doi.org/10.1177/001857870403900608). The zones of inhibition were measured and the interpretation (S - sensitive, I - intermediate, R - resistant) was drawn as per zone size interpretative chart provided by the manufacturer.


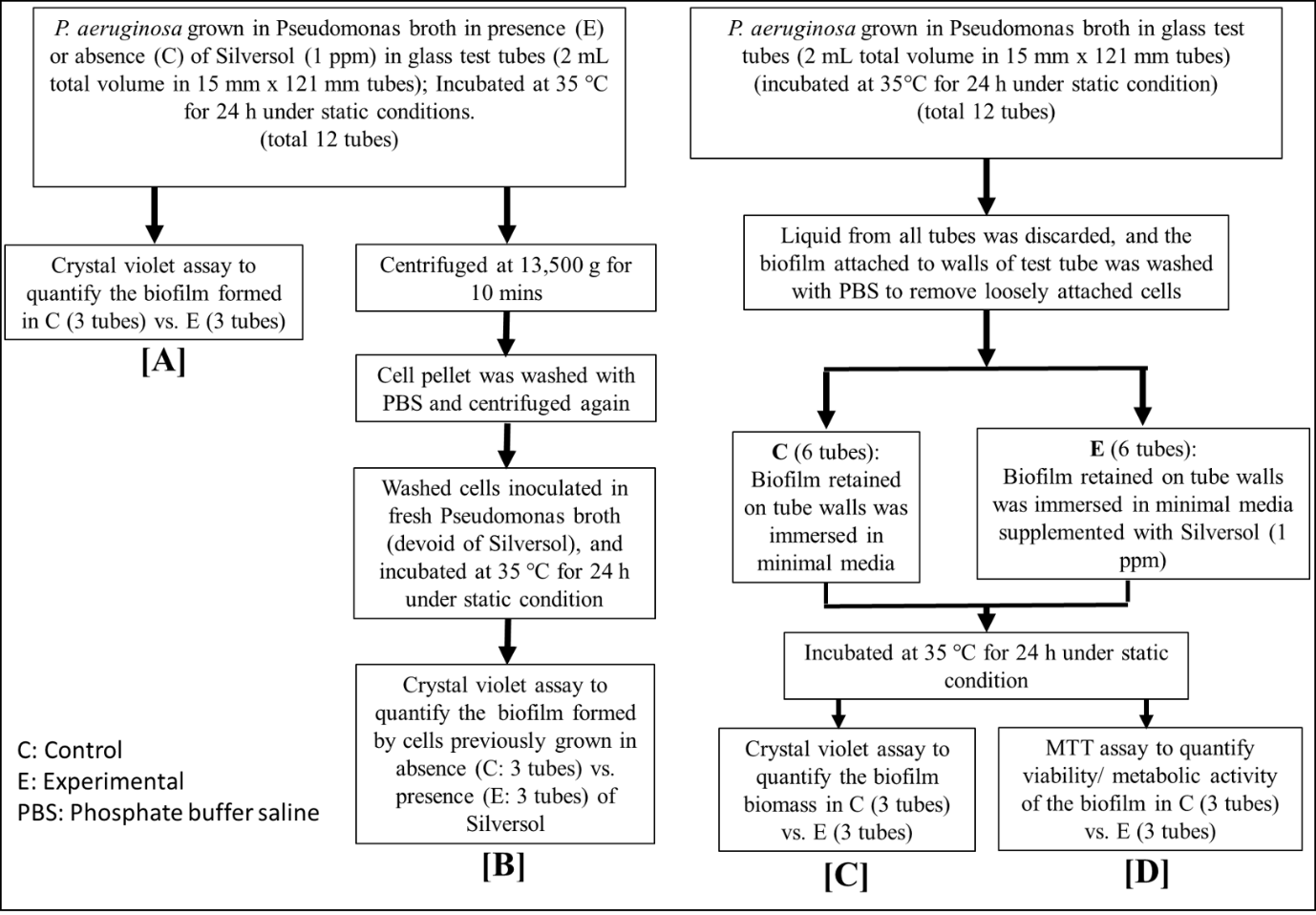


**Figure S2: Flowchart depicting schematic of all biofilm assays**

**(A)**Quantification of biofilm formation in presence or absence of Silversol; **(B)** Quantification of biofilm formation by silver-pre-treated vs. non-pre-treated *P. aeruginosa* cells; **(C)** Quantification of biofilm eradication after adding Silversol onto pre-formed biofilm; **(D)** Quantification of Silversol’s effect on metabolic activity of pre-formed biofilm

**
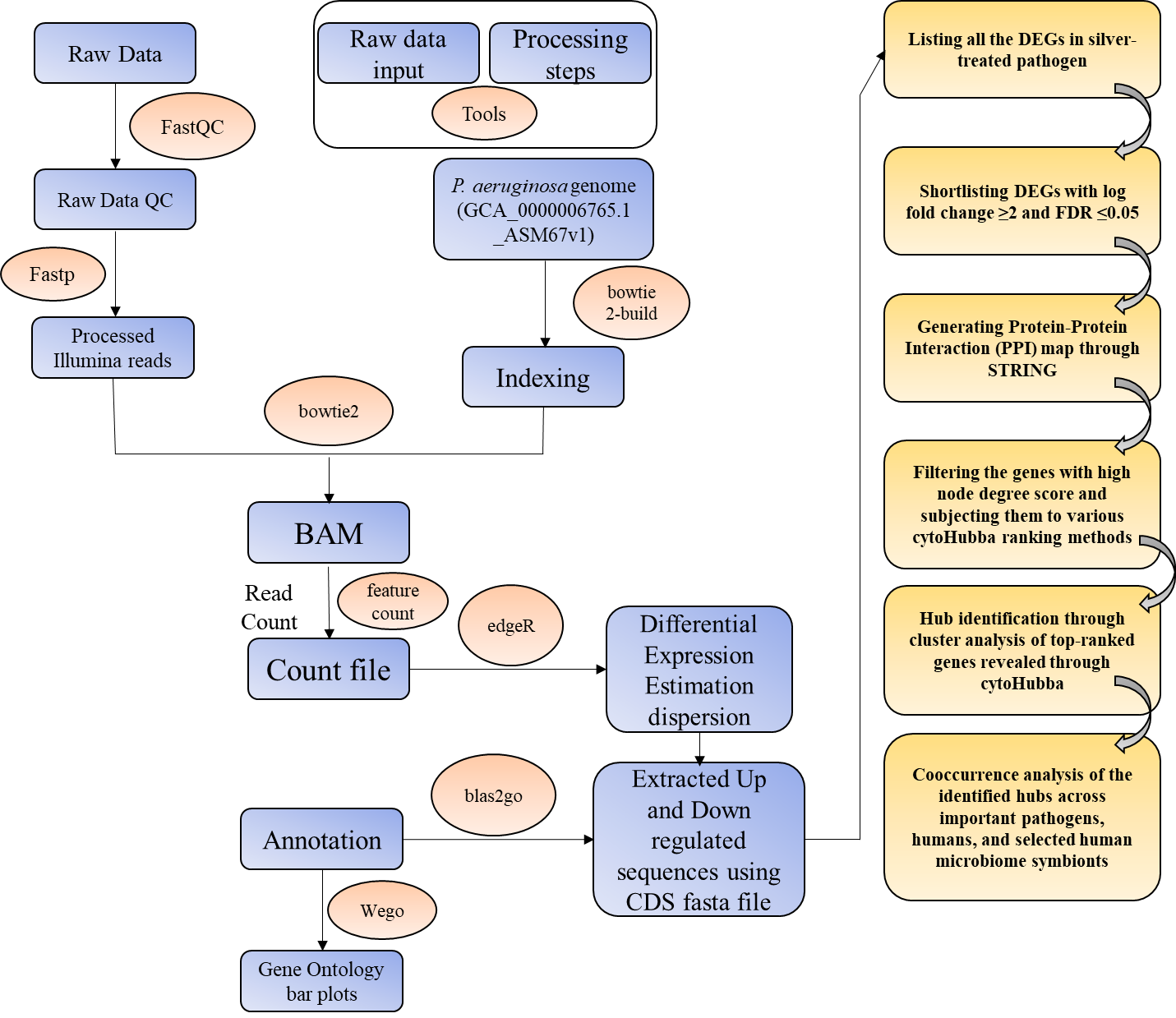
Figure S3: A schematic presentation of the methodology/workflow employed for whole transcriptome and network analysis**

DEG: Differentially Expressed Genes

**Table S2.** **Quantification of extracted RNA, library, and insert size**

| **Sr. no.** | **Sample name** | **Quantification of extracted RNA** | | | **Library quantification and insert size analysis** | |
| --- | --- | --- | --- | --- | --- | --- |
|  |  | **OD_260_/OD_280_** | **OD_260_/OD_230_** | **RIN value** | **ng/ µL** | **Insert size** |
| 1 | Control | 2.15 | 2.47 | 6.5 | 21 | 149,324,439 |
| 2 | Experimental | 2.14 | 2.95 | 8.3 | 24.4 | 366,439 |

**Table S3. Temperature profile for RT-PCR assay**

| **Temperature** (°C) | **Time**  (s) | **Remarks** |
| --- | --- | --- |
| PCR cycles (45 cycles) | |  |
| 95 | 15 | Denaturation temperature |
| 59 | 60 | Annealing temperature |
| **Melt curve stage** | |  |
| 95 | 15 |  |
| 60 | 60 |  |
| 95 | 15 |  |

**
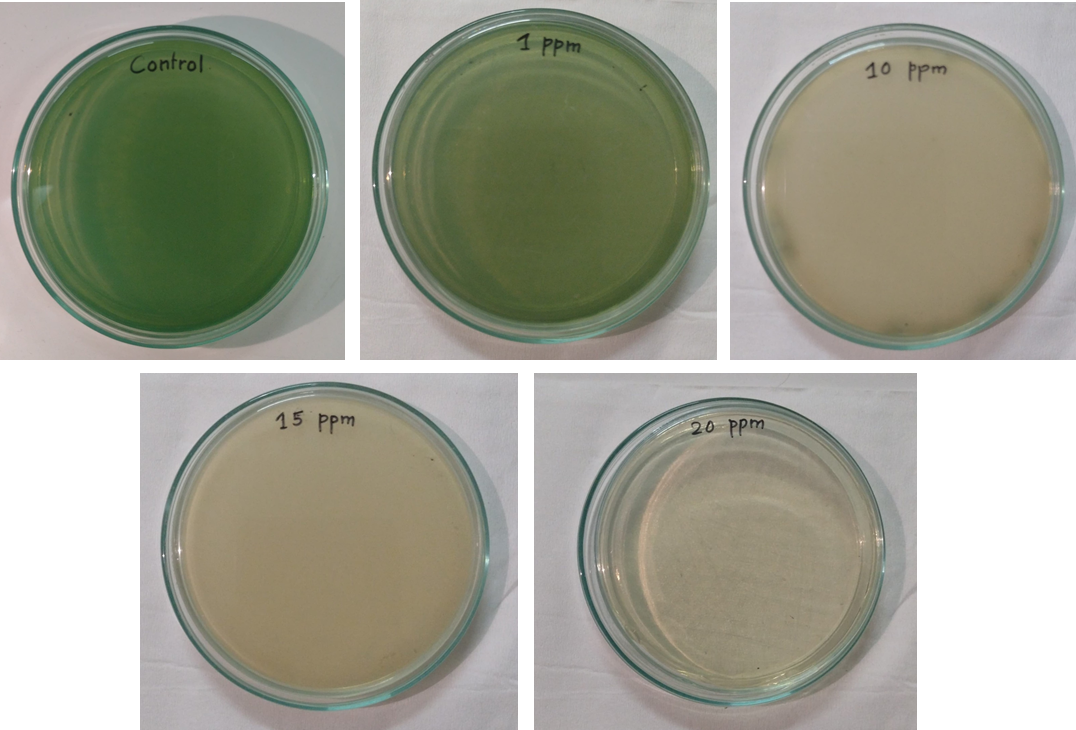
**

**Figure S4. MBC of Silversol****^®^ against *P. aeruginosa* was between 16-20 ppm**

Cells grown in presence of Silversol**^®^** were subsequently plated onto Pseudomonas agar. Those coming from 10-15 ppm silver tubes displayed non-pigmented growth, while those from 20-ppm failed to give rise to any visible growth.


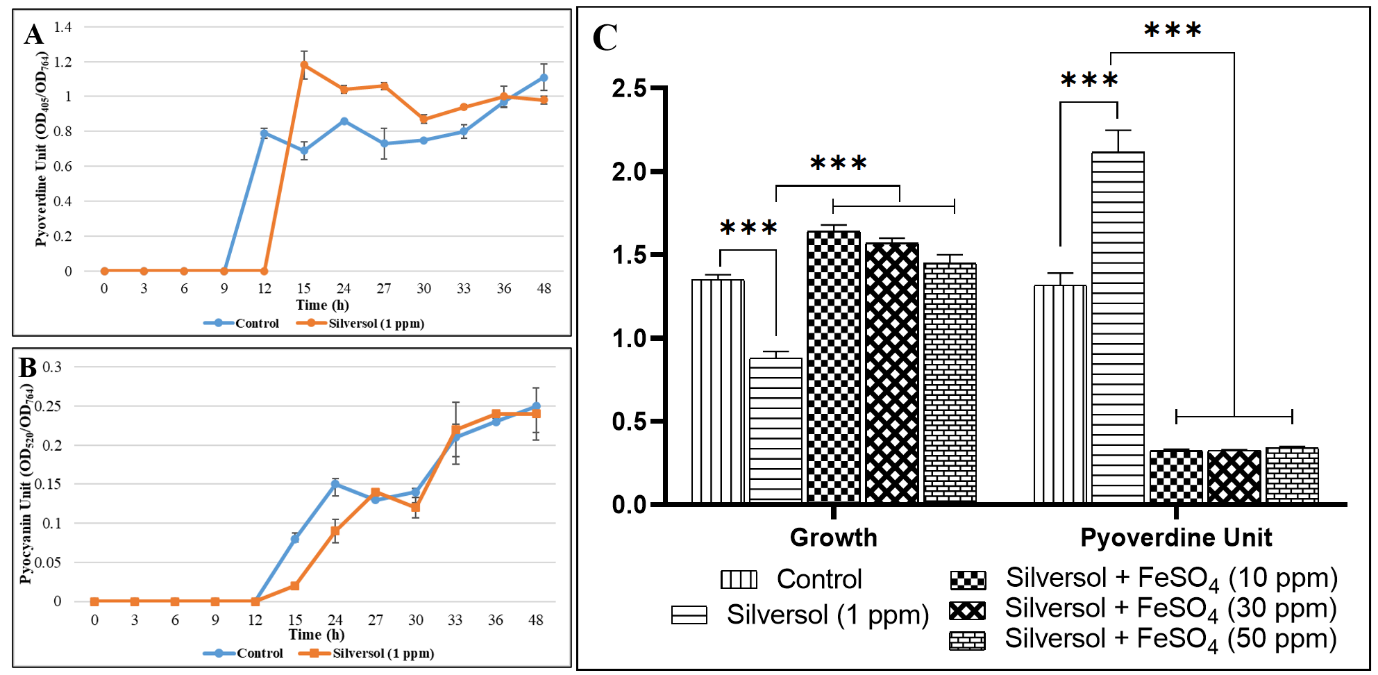
**Figure S5. Silversol’s growth- and pigment- modulatory effect on *P. aeruginosa***

(A)-(B): Silversol delays onset of pigment production in *P. aeruginosa*, but over extended period of incubation, the bacterium is able to overcome this pigment-inhibitory effect of sub-lethal concentration of Silversol. (C): Iron supplementation can protect *P. aeruginosa* from Silversol’s effect on growth and pyoverdine production. Presence of additional iron (in form of FeSO_4_) in growth media does not allow Silversol to inhibit bacterial growth or force excess siderophore (pyoverdine) production.

**
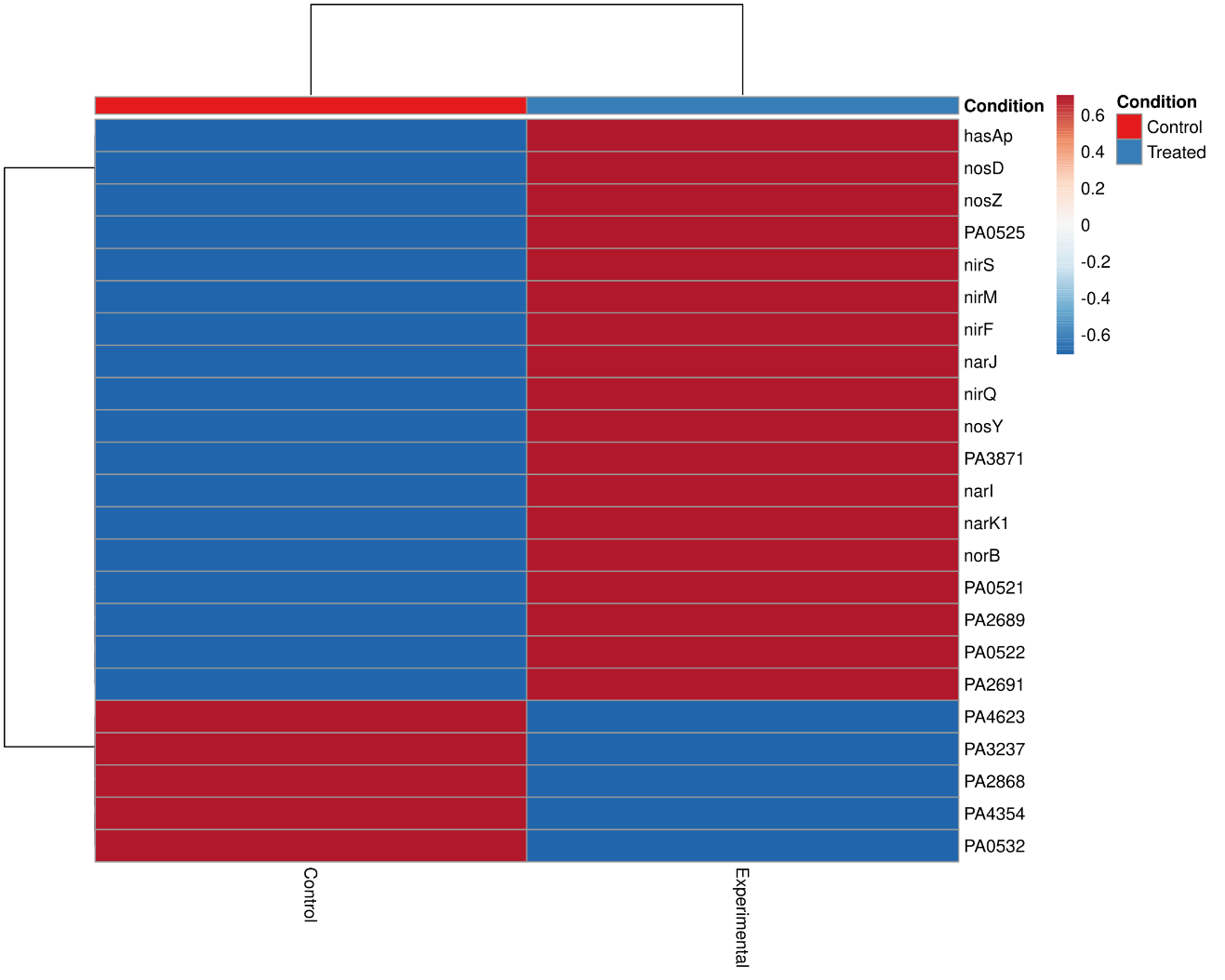
**

**Figure S6. Heat map of DEGs in Silversol^®^-exposed *P. aeruginosa***

Heat map generated using the online software tool ClustVis (https://biit.cs.ut.ee/clust vis/) showing upregulated and downregulated genes with FDR<0.05 and log fold change ‘± 2’. Values plotted are means of three replicates.

**
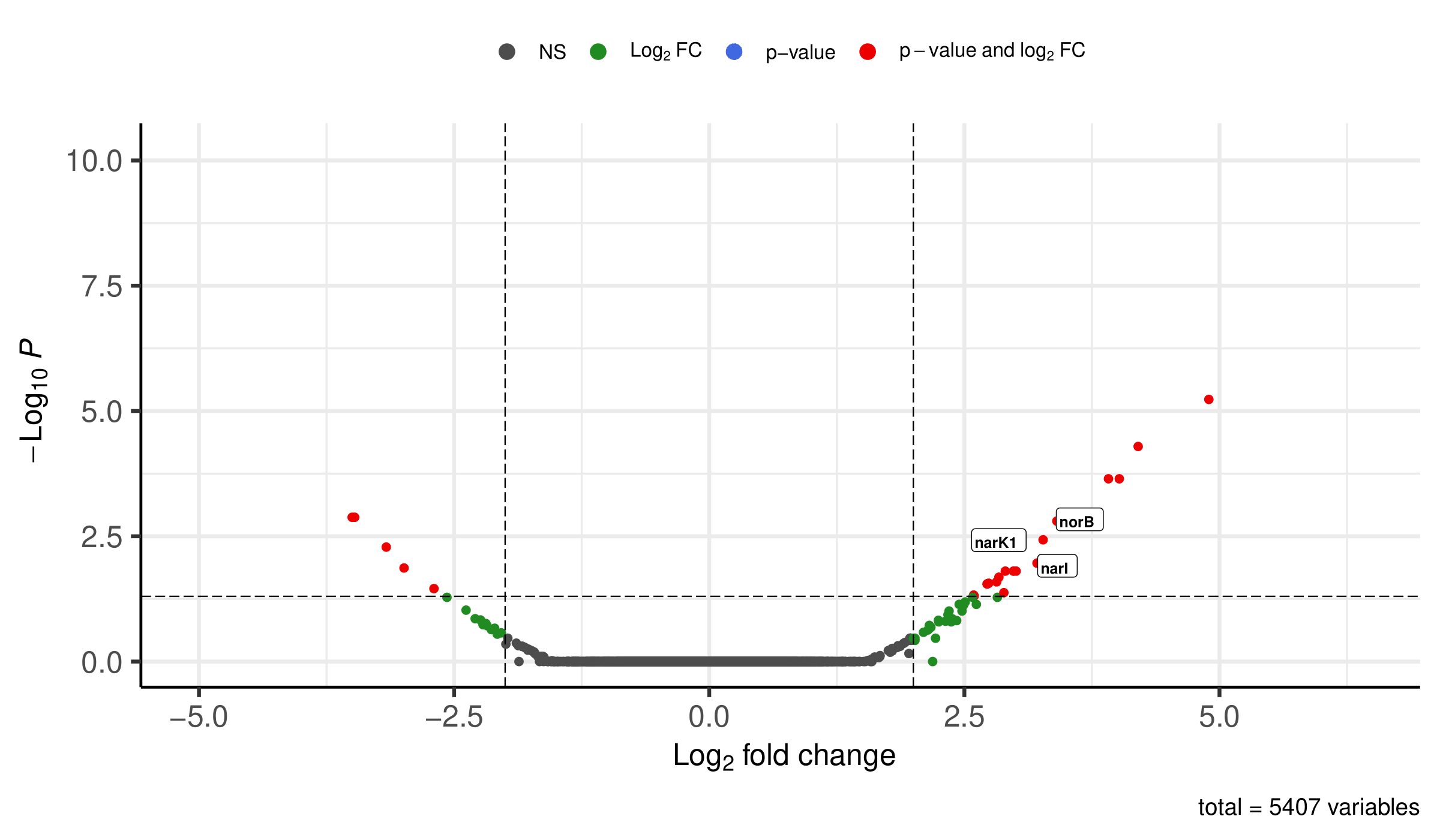
Figure S7. Volcano Plot of experimental versus control samples**

Volcano plot of expressed genes of experimental culture compared to control culture. The y- axis illustrates −log 10 p values, and the x-axis corresponds to a log 2-fold change of gene expression between both cultures. The red points represent differently expressed genes satisfying the dual criteria of FDR< 0.05 and log fold change ≥2.

**
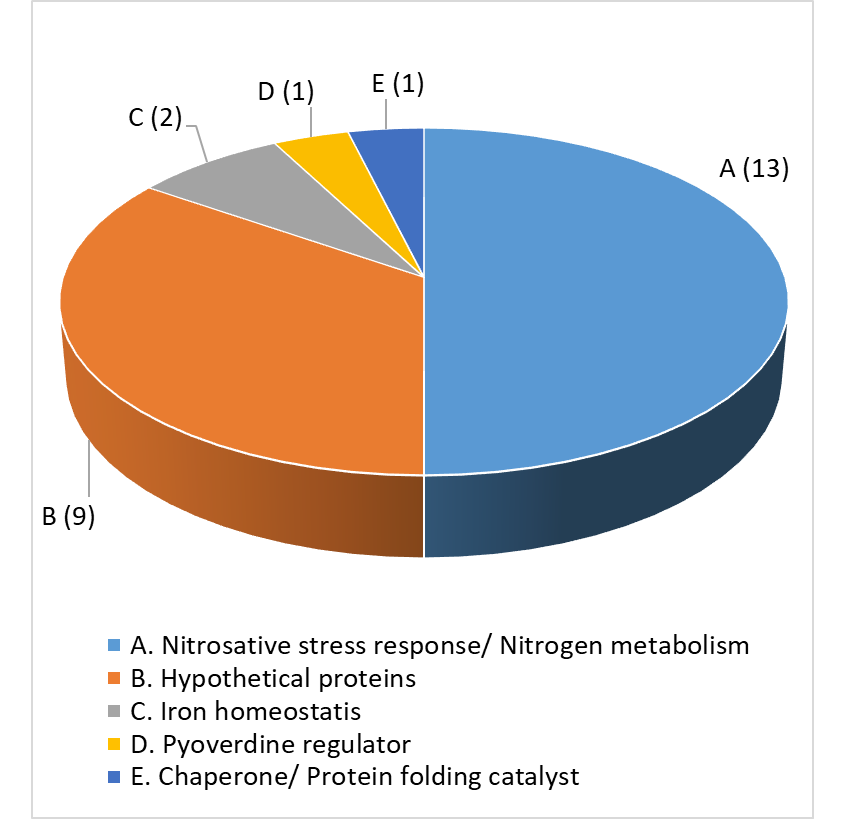
**

**Figure S8. Function-wise categorization of the significantly differentially expressed genes in Silver-treated *Pseudomonas aeruginosa.*** Figures in parentheses indicate number of genes in that particular category.
